# Lipid Nanoparticles Traverse Non-Corneal Path to Reach the Posterior Eye Segment: In Vivo Evidence

**DOI:** 10.3390/molecules26154673

**Published:** 2021-08-02

**Authors:** Carmelo Puglia, Debora Santonocito, Giuseppe Romeo, Sebastiano Intagliata, Giovanni Luca Romano, Enrica Strettoi, Elena Novelli, Carmine Ostacolo, Pietro Campiglia, Eduardo Maria Sommella, Rosario Pignatello, Claudio Bucolo

**Affiliations:** 1 Department of Drug and Health Science, University of Catania, Viale Andrea Doria 6, 95125 Catania, Italy; debora.santonocito@unict.it (D.S.); gromeo@unict.it (G.R.); s.intagliata@unict.it (S.I.); rosario.pignatello@unict.it (R.P.); 2 NANO-i—Research Centre on Ocular Nanotechnology, University of Catania, 95125 Catania, Italy; 3 Department of Biomedical and Biotechnological Sciences, Section of Pharmacology, University of Catania, 95125 Catania, Italy; giovanniluca.romano@unict.it (G.L.R.); claudio.bucolo@unict.it (C.B.); 4 Center for Research in Ocular Pharmacology—CERFO, University of Catania, 95125 Catania, Italy; 5 CNR Neuroscience Institute, Area della Ricerca, 56124 Pisa, Italy; enrica.strettoi@in.cnr.it (E.S.); novelli@in.cnr.it (E.N.); 6 Department of Pharmacy, University of Naples Federico II, 80131 Naples, Italy; ostacolo@unina.it; 7 Department of Pharmacy, University of Salerno, 84084 Fisciano, Italy; pcampiglia@unisa.it (P.C.); esommella@unisa.it (E.M.S.)

**Keywords:** ocular drug delivery, nanomedicine, fluorescent nanoparticle, fluorescence microscopy, retina

## Abstract

Lipid-based nanocarriers (LNs) have made it possible to prolong corneal residence time and improve the ocular bioavailability of ophthalmic drugs. In order to investigate how the LNs interact with the ocular mucosa and reach the posterior eye segment, we have formulated lipid nanocarriers that were designed to bear a traceable fluorescent probe in the present work. The chosen fluorescent probe was obtained by a conjugation reaction between fluoresceinamine and the solid lipid excipient stearic acid, forming a chemically synthesized adduct (ODAF, *N*-(3′,6′-dihydroxy-3-oxospiro [isobenzofuran-1(3*H*),9′-[9*H*] xanthen]-5-yl)-octadecanamide). The novel formulation (LN-ODAF) has been formulated and characterized in terms of its technological parameters (polydispersity index, mean particle size and zeta potential), while an in vivo study was carried out to assess the ability of LN-ODAF to diffuse through different ocular compartments. LN-ODAF were in nanometric range (112.7 nm ± 0.4), showing a good homogeneity and long-term stability. A TEM (transmission electron microscopy) study corroborated these results of characterization. In vivo results pointed out that after ocular instillation, LN ODAF were concentrated in the cornea (two hours), while at a longer time (from the second hour to the eighth hour), the fluorescent signals extended gradually towards the back of the eye. From the results obtained, LN-ODAF demonstrated a potential use of lipid-based nanoparticles as efficient carriers of an active pharmaceutical ingredient (API) involved in the management of retinal diseases.

## 1. Introduction

The retina, together with the ciliary body, the choroid and iris represent the posterior segment of the eye [1]. It is the site of many ocular diseases, such as diabetic retinopathy, age-related macular degeneration and optic neuropathy [2,3]. The treatment of these posterior eye segment diseases remains a great challenge. The efficacy of topical administration into the eyes by conventional eye drops is limited by numerous pre-corneal drug removal mechanisms, which oppose trans-corneal drug absorption [1] and physiological barriers [4,5]; in fact, the ocular bioavailability of topical drugs is less than 5–10%. Therefore, frequent instillations of eye drops are necessary to achieve the awaited therapeutic effect [6]; this, especially in chronic therapy, decreases patient compliance [7]. For this reason, conventional therapy for the treatment of posterior eye segment diseases needs administration by the transcleral/periocular route, which includes peribulbar, retrobulbar and subconjunctival injections or the application of ocular implants. These therapies show numerous disadvantages, as they require high costs [8], involve an invasive procedure and can induce serious ocular complications [9].

Nowadays, there is an increasing need to find a therapy for retinal diseases, such as diabetic retinopathy, age-related macular degeneration and optic neuropathy. 

Recently, the development of lipid-based nanocarriers (LNs) has made possible to prolong corneal residence time and improve the local bioavailability of ophthalmic drugs [10,11,12,13]. These systems possess important advantages for ocular application, such as controlled drug release, high drug loading, good bioavailability and excellent tolerability [1,14,15]. In particular, they are able to improve the interaction with the ocular mucosa, thus producing an increase in the ocular bioavailability [16]. Recent studies demonstrated that LNs could be useful for ocular application [1,17,18,19].

Yadav and coworkers formulated solid lipid nanoparticles loaded with atorvastatin (ATS-SLNs) as eye drops for the management of age-related macular degeneration (AMD) [18]. The encapsulation into lipid nanocarriers demonstrated that ATS-SLN was 8 and 12 times more bioavailable (AUC) in aqueous and vitreous humor, respectively, than free ATS. 

Recently, Chetoni and coworkers demonstrated the importance of the nanotechnology approach in the ophthalmic field [19]. The researchers formulated lipid nanoparticles that were able to modify the pharmacokinetic parameters and the tobramycin distribution in the ocular tissues; Tobra-SLN produced a concentration of tobramycin in the aqueous humor twofold and fivefold higher, compared to those obtained from the tobramycin solution after one hour and three hours, respectively [19].

In order to investigate how LNs interact with the ocular mucosa and reach the posterior eye segment, we have formulated nanocarriers designed to bear a traceable fluorescent probe in the present work. This probe was obtained by a conjugation reaction between fluoresceinamine and the solid lipid excipient stearic acid, forming a stable covalent adduct (ODAF, *N*-(3′,6′-dihydroxy-3-oxospiro[isobenzofuran-1(3*H*),9′-[9*H*]xanthen]-5-yl)-octadecanamide). The fluorescent lipid was then nanostructured to obtain LN-ODAF, which was characterized in terms of technological parameters (polydispersity index, mean particle size and zeta potential), while an in vivo study was carried out to assess the ability of LN-ODAF to diffuse through the different ocular compartments.

## 2. Results

### 2.1. ODAF Synthesis and Characterization 

**Figure 1:**
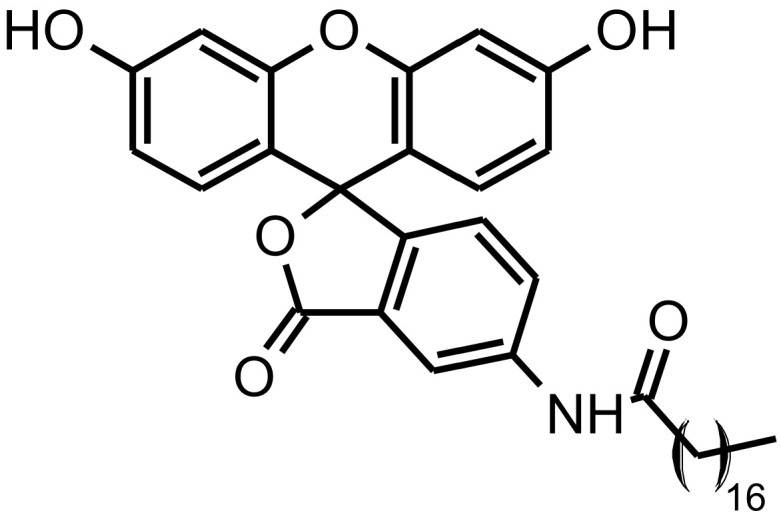
ODAF structure (lactone form).

*N*-(3′,6′-dihydroxy-3-oxospiro[isobenzofuran-1(3*H*),9′-[9*H*]xanthen]-5-yl)-octadecanamide (5-*N*-octadecanoyl-aminofluorescein, ODAF) is a known lipophilic fluorescein derivative, used as a fluorescent label for a number of applications [20,21] (Figure 1).

In this work, with the aim to get ODAF ready to produce enough amounts to formulate the LN-ODAF formulation, we performed its synthesis and characterization ex novo.

Synthesized ODAF (lactone form) was spectroscopically characterized by ^1^H-NMR (Figure S1, Supplementary Materials), ^13^C-NMR (Attached Proton Test, APT; Figure S2), 2D ^1^H-, ^13^C-NMR (gHSQCAD experiment; Figure S3) and MALDI-TOF mass technique; analyses of all acquired spectra confirmed the ODAF structure. In particular, formation of octadecanamide from 5-aminofluorescein and stearic acid was supported by signals, as the triplet at 0.83 δ, multiplets at 1.10–1.40 and 1.56–1.66 δ and the triplet at 2.35 δ in the ^1^H-NMR spectrum. This was attributable to the aliphatic hydrogens of amide chain, and by signals in the APT spectrum in the range 13.93–36.48 δ for all the seventeen carbons of the aliphatic chain, along with that at 171.92 δ for the carbon atom of the amide group. 

Bidimensional gHSQCAD spectrum (see SI), where all the expected ^1^H and ^13^C correlations were present, further confirmed the ODAF structure.

### 2.2. Formulation and Characterization of LN-ODAF

LN-ODAF were formulated by the solvent-diffusion technique, using ODAF as the lipid phase. The mean particle size, PDI and zeta potential were measured by dynamic light scattering (DLS) analysis. As reported in Table 1, LN-ODAF showed small mean particle sizes of 110 nm and a good homogeneity. The Z potential value of −22 mV predicted good storage stability of the formulation. 

**Figure 2:**
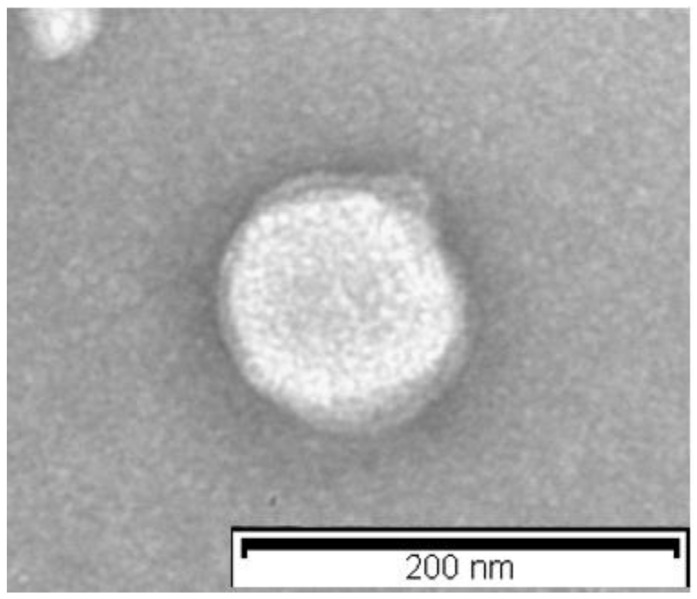
Transmission electron microscopy images of LN-ODAF. The scale bar represents 200 nm.

The morphology of the lipid nanoparticles was determined using transmission electron microscopy (TEM). The TEM image showed that the lipid nanoparticles had a spherical appearance, having a particle size around 110 nm (Figure 2). This particle size was in agreement with DLS data.

### 2.3. Chemical Stability of ODAF

The chemical stability of ODAF (50 mg/mL) was monitored at two different time points, 2 and 4 h: in HCOONH_4_ 5 mM solution, prepared in MeOH 70%, adjusted to pH 5 with HCOOH; in HCOONH_4_ 5 mM solution, prepared in MeOH 70%, at pH 7; and in Na_2_CO_3_ solution, prepared in MeOH 70%, at pH 9. Values are expressed as area % of ODAF in the different conditions, compared to the area of ODAF solubilized in methanol (Table 2).

The increase in area percentage at pH 9 was due to a sensitivity of the probe to pH conditions, which increases its fluorescence at basic pH. At the same time, the increase in area at four hours was due to an increase in the solubility of the sample over time. In fact, especially at basic pH, the molecule had a poor solubility in the water/methanol solution used. Again, free fluoresceinamine was never revealed, indicating the complete chemical stability of the adduct.

### 2.4. In Vivo Study 

#### Ocular Distribution 

The analytical method was standardized for the quantification of ODAF and fuoresceinamine alone in ocular tissues (Table 3). No trace of fluoresceinamine was ever detected in any of the samples, although the substance lent itself very well to analysis, with extremely low LoD and LoQ values (in the order of femtomolar).

A bright green fluorescence, visible with the Zeiss cube N3, specific for FITC, was detected in all the ocular sections obtained upon fluorescent LNs administration, including those from the mouse sacrificed as early as two hours afterwards. Specific fluorescence was absent from control eyes receiving non-fluorescent nanoparticles, and from the eyes of untreated, control mice. 

**Figure 3:**
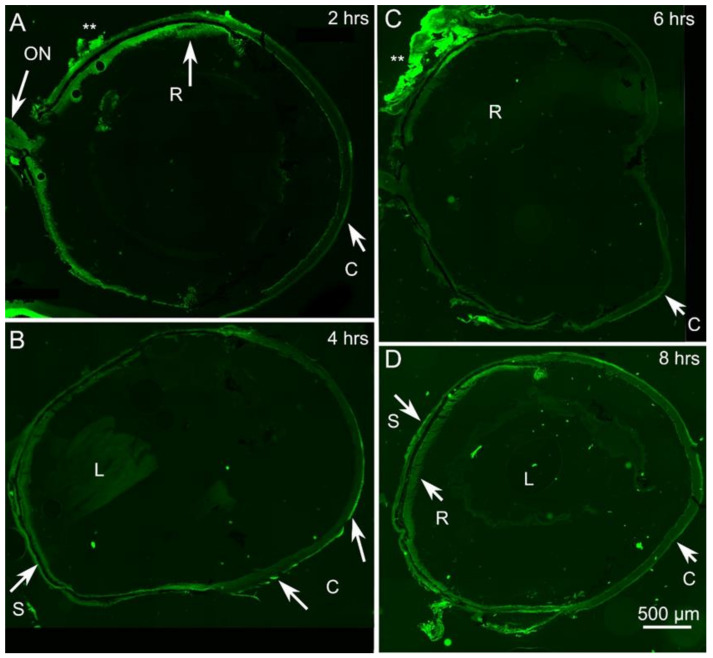
LN-ODAF diffusion to ocular structures. (**A**–**D**) Progressive invasion of the green fluorescence signal in time. C: cornea; ON: optic nerve; R: retina; S: sclera. L: Lens. Asterisks in (**A**,**C**) indicate periocular structures abutting areas of bright fluorescent staining of the underlying retina, suggesting outer-to-inner diffusion at focal points.

Two hours after instillation, the superficial layers of the cornea were brightly fluorescent, although not continuously along the corneal surface (Figure 3A). A weak fluorescence was detectable at some retinal locations. At longer times (four and six hours, Figure 3B,C), the fluorescent signals extended gradually toward the back of the eye and encompassed the sclera. Scattered, periocular structures (asterisks in Figure 3C) showed specific and intense fluorescence. The retina abutting these structures was clearly labelled as well. Eight hours post administration (Figure 3D), multiple organs appeared diffusely labelled, including components of the ciliary body. To note, corneal fluorescence persisted for the whole time interval considered here, becoming gradually more spread. Conversely, no signal was detected in the aqueous body, lens and vitreous body using fluorescence microscopy. No specific signal was evident in non-treated, control eyes.

Therefore, microscopy observation supported the notion that the diffusion of LN-ODAF had begun at the corneal level, and spread to the back of the eye to reach the sclera and the retina.

## 3. Discussion

LN-ODAF were prepared by the solvent-diffusion technique, which proved to be a valid and highly reproducible method for this compound, minimizing the use of organic solvent, which might cause toxicity concerns if residues of it remain [22,23]. Preliminary studies have been directed to establish the best concentration of ODAF to be used in order to obtain nanotechnological parameters suitable for ocular administration. All of the ingredients used in the preparation of LN-ODAF are claimed to be safe for ocular use [1,24,25], and they did not produce any toxicity issues in animals during the study (data not shown). This result is in accordance with data reported in recent published studies [17]. In fact, many examples are reported regarding the safety of the surfactant Lutrol^®^ F68, widely used in LN formulation, demonstrating a full biocompatibility with the ocular tissues [14].

In general, the nanoparticle approach permits a state of matter, characterized by a higher and greater surface area available for association between the cornea and conjunctiva. Furthermore, the achievement of nanosized particles will help the passage across the anatomical constraints in the eye, and better precorneal retention. In a recent manuscript from our group, this approach was used to enhance the ocular bioavailability of palmitoylethanolamide (PEA), an endogenous congener of the endocannabinoid anandamide (AEA) with a potent anti-inflammatory activity, which can be exploited in different pathological conditions and in a variety of biological systems including the retina [16]. In particular, PEA based lipid nanoformulation (PEA-NLC) was administered into rabbit eye, and the drug pharmacokinetic profile was evaluated. Interestingly, the retinal levels of PEA were significantly higher in the group treated with PEA-NLC formulation versus aqueous suspension of PEA, but the mechanism by which the drug may be delivered to the posterior segment from the pre-corneal area was not clear. The evidence indicates that PEA permeation may occur via the conjunctival/scleral route, followed by distribution to the choroid, vitreous and retina, although the corneal route cannot be excluded. In a more recent study, the same formulation was able to significantly inhibit retinal tumor necrosis factor-α (TNF-α) levels in streptozotocin-induced diabetic rats [26]. Again, pharmacokinetics data showed that PEA was able to reach the back of the eye once enclosed in NLC, but no evidence on the drug delivery mechanism was determined.

In the present work, LN-ODAF, a lipid fluorescent nanocarrier was formulated in order to investigate how LNs interact with the ocular mucosa and reach the posterior eye segment. The results from the microscopy observation outlined that the diffusion of LN-ODAF begins at the corneal level, and spreads toward the back of the eye to reach the sclera and the retina. This evidence seems to suggest a scleral absorption, and the hypothesis is supported by the absence of a fluorescent signal in the aqueous body, lens and vitreous body.

Amrite and colleagues demonstrated that the arrangement of nanoparticles after transcleral administration is size dependent [27,28]. An in vitro study on nanoparticles with sizes of 20 and 200 nm showed that drug administration in the retina was affected by the clearance of the particles. The smaller (20 nm) nanoparticles were able to cross the sclera, and were rapidly eliminated due to the periocular circulation (blood and lymphatic); therefore, no ocular effect was observed. Instead, larger nanoparticles (200 nm) were unable to cross the sclera or sclera-choroid-retinal pigment epithelium (RPE), and persisted in the periocular site for at least two months. Therefore, larger nanoparticles (200 nm) better sustained the retinal drug delivery compared to smaller ones. Another study showed the intraocular kinetics of different sized nanocarriers (2 μm, 200 nm and 50 nm) after the injection into the vitreous cavity [29]. Histological results revealed that nanocarriers with a diameter of 2 μm were observed in the vitreous cavity and trabeculae, while those with a diameter of less than 200 nm were seen in the retina. Therefore, the nanocarrier size plays a crucial role in the drug delivery to the target tissue. In the present study, LN-ODAF particle size distribution showed that most of the portion of LNs were found between 50–150 nm, hence, it can be concluded that their average particle size of 112.7 nm suited their capacity for retinal targetability.

The rigidity of the lipid matrix of the nanoparticles represents another possible explanation of the ocular biovailability of LN-ODAF in the back of the eye.

Fluorometric investigation showed that fluorescent liposomes formulated with L-α-distearoylphosphatidylcholine had a higher luminescence intensity in the retinal tissue, than those composed of egg phosphatidylcholine. This was closely related to the stiffness and size of the liposomes [30]. It seems that rigidity maintained the stability of the carrier system in the ocular environment, and, therefore, LNs made of lipids solid in nature, could exhibit this feature.

Notwithstanding the lipophilic characteristic of LN-ODAF, which should in theory reduce its permeability through the sclera, we thought that the presence of an outer surfactant coating (poloxamer 188) could endow them with hydrophilic properties, thus improving their transcleral absorption. Another important feature of LN-ODAF is represented by their chemical stability. In fact, the results obtained have demonstrated that the observed fluorescence in the ocular tissues is referred to the whole nanocarrier and not to the release of fluorescinamine.

The results of the present work clearly need to be studied in depth, although they gave us important indications regarding the mechanism involved in ocular drug delivery via lipid-based nanoparticles. The next step of the present research could be represented by the formulation of LN-ODAF, loaded with an active pharmaceutical ingredient (API) for the treatment of an ocular disease, in order to evaluate not only the ocular distribution of the vehicle in the eye sections but also the pharmacological effect due to the carried API.

## 4. Materials and Methods

### 4.1. Materials

Lecinol S-10, hydrogenated lecithin, was obtained from Nikko Chemical (Milan, Italy), and Lutrol F68^®^ (MW 8400 g/mol) was provided by BASF Chem-Trade GmbH (Burgbernheim, Germany). Stearic acid (MW 284.48 g/mol), 5-aminofluorescein (MW 347.32 g/mol), (hydroxypropyl)methyl cellulose and all reagents were LCMS grade and purchased from Merck (Darmstadt, Germany).

### 4.2. Synthesis and Characterization of ODAF, N-(3′,6′-dihydroxy-3-oxospiro[isobenzofuran-1(3H),9′-[9H] xanthen]-5-yl)-Octadecanamide

A mixture of stearic acid (0.102 g, 0.33 mmol) and 5-aminofluorescein (0.106 g, 0.30 mmol) was stirred at room temperature in dry tetrahydrofuran (20 mL). TLC was used to monitor the progress of the reaction. After three hours, stirring was stopped and the solid was collected by filtration under a vacuum and dried. The obtained product was purified by flash column chromatography, using a mixture of ethyl acetate, cyclohexane and acetic acid (5:5:0.02, *v*/*v*/*v*) as eluent. The homogeneous fractions were collected, and the solvent was removed under reduced pressure to yield a pure orange powder (0.084 g, 46%). The melting point (Mp) was 213–216 °C.

Melting points were determined in an IA9200 electrothermal apparatus, equipped with a digital thermometer in glass capillary tubes and were uncorrected. The ^1^H-NMR and ^13^C-NMR spectra of ODAF were determined with a Varian Inova Unity (500 MHz) spectrometer using a DMSO-d6 solution. The chemical shifts are given in δ values (ppm), using tetramethylsilane (TMS) as the internal standard; the coupling constants (*J*) are given in hertz (Hz). The signal multiplicities are characterized as s (singlet), d (doublet), t (triplet) or m (multiplet). MALDI-TOF mass spectra were collected by a Voyager DE (PerSeptive Biosystem, BioSurplus, Inc., San Diego, CA, USA), using a delay extraction procedure (25 kV applied after 2600 ns with a potential gradient of 454 V·mm^−1^ and a wire voltage of 25 V) and detecting the ions in a linear mode. Thin-layer chromatography (TLC) on Merck plates (aluminum sheet coated with silica gel 60 F254) was used to monitor the progress of the reaction; spots were visualized under UV (λ = 254 and 366 nm). ODAF purification by column chromatography was performed using Merck silica gel 60 (230–400 mesh).

^1^H-NMR (500 MHz, DMSO-d6): δ 10.31 (s, 1H), 10.09 (s, 2H), 8.31 (d, *J* = 1.9 Hz, 1H), 7.81 (dd, *J* = 1.9 Hz, *J* = 8.3 Hz 1H), 7.17 (d, *J* = 8.3 Hz, 1H), 6.65 (d, *J* = 2.3 Hz, 2H), 6.56 (d, *J* = 8.7 Hz, 2H), 6.54 (dd, *J* = 2.3 Hz, *J* = 8.7 Hz, 2H), 2.35 (t, *J* = 7.3 Hz, 2H), 1.66–1.56 (m, 2H), 1.40–1.10 (m, 28H), 0.83 (t, *J* = 6.9 Hz, 3H).

^13^C-NMR (126 MHz, DMSO-d6): δ 171.92, 168.64, 159.43, 151.90, 146.56, 140.87, 129.03, 126.92, 126.21, 124.31, 113.32, 112.52, 109.76, 102.20, 83.01, 36.48, 31.29, 29.03, 28.92, 28.85, 28.78, 28.70, 28.62, 25.04, 22.09, 13.93.

MALDI-TOF (*m*/*z*): 636.9 [MNa^+^], 615.0 [MH^+^]; calculated for C_38_H_47_NO_6_: 613.78.

### 4.3. Preparation of LN-ODAF

LN-ODAF were prepared by a solvent-diffusion technique [22], using ODAF as the lipid phase and Lutrol F68^®^ (Poloxamer 188) as the surfactant.

ODAF (0.0115 g) was solubilized in ethanol (2.3 mL) and maintained in a fluid state at 50 °C. The aqueous phase was constituted by hydroxypropylmethyl cellulose (0.115 g), soy lecithin (0.115 g), Lutrol F68^®^ (0.115 g) and distilled water (11.5 mL). Lipid melted phase was slowly dispersed into the hot aqueous surfactant solution (50 °C), by using a high-speed stirrer (Ultra-Turrax T25, IKA-Werke GmbH &Co. Kg, Staufen, Germany) at 15,000 rpm for eight minutes. The obtained coarse emulsion was ultrasonified by using an ultrasonic processor (UP 400 S, Dr. Hielscher GmbH, Stuttgart, Germany) for ten minutes. Then the hot nanoemulsion was dipped in an ice bath for five minutes to obtain LN-ODAF. Finally, the organic solvent was removed by vacuum.

### 4.4. Characterization of LN-ODAF

Submicron particle size and polydispersity index (PDI) analysis were performed using a Zeta Sizer Nano-ZS90 (Malvern Instrument Ltd., Worcs, England) equipped with a 5 mW solid-state laser with a wavelength of 670 nm. Measurements were performed at 20 ± 0.2 °C at an angle of 90°. The zeta potential (ZP, ξ) was measured by electrophoretic light scattering (ELS) using the same instrument. The measurements were recorded at 25 °C, using three sets of measures up to 100 to achieve an average value. Both analyses were carried out on samples previously diluted with bidistilled water in a 1:10 *v*:*v* ratio. Each value was measured at least in triplicate.

Morphological characteristics of LN-ODAF were investigated using TEM (Philips EM 400T microscope, Eindhoven, The Netherlands). TEM samples were prepared by deposition of diluted (100-fold) LN-ODAF formulation onto an aluminum specimen stub covered with a double-sided adhesive carbon disk. After water vaporization, the samples were spray coated with chromium prior to imaging (Quorum Q150T ES East Grinstead, West Sussex, UK).

### 4.5. In Vivo Study

#### 4.5.1. Animals

Animal experiments were in accordance with European directives no. 2010/63/UE and Italian directives D.lgs. 26/2014, and complied with the statements of Association for Research in Vision and Ophthalmology (ARVO) for the use of animals in ophthalmic and visual research (Protocols approval #14/D, 07/14/2014) and by the ethical committee of the CNR Neuroscience Institute (Pisa, Italy). New Zealand rabbits (weight 2.0–2.5 kg) and C57Bl6/J from 6–8-week-old male mice were purchased from Charles River (Calco, Italy). Animals were housed in standard conditions, with free access to food and water, in a light-controlled room at a controlled range of temperature and humidity.

#### 4.5.2. Ocular Pharmacokinetics

Rabbit eyes (*n* = 16) received single ocular topical administration (30 μL) of LN formulation. Animals were sacrificed by intravenous administration of 0.3 mL/kg of Tanax^®^ (Intervet, Milan, Italy), after sedation with an intramuscular administration of 10 mg/kg of Zoletil^®^ (Virbac, Milan, Italy). Animals were sacrificed at one, three, eight and sixteen hours after ocular topical administration (30 μL) of LN-ODAF formulation. Eyes were enucleated and ocular tissues (cornea, sclera, aqueous, iris, lens, vitreous, and retina) were then collected. Tissue samples were stored at −80 °C until quantitative analysis.

#### 4.5.3. ODAF Quantitation in Ocular Tissues

##### Extraction

Samples were stored at −80 °C before use. Aqueous and vitreous humors were filtered by 0.22 μM Phenex PTFE filters (Phenomenex, Castel Maggiore, BO, Italy) and directly analyzed by UHPLC-MS/MS. The lens, retina, iris, sclera and cornea were brought to a temperature of −55 °C and lyophilized overnight. The resulting powders were weighed, carefully crushed in agate mortar and extracted. Extractions were performed by adding 1 mL of frozen methanol to 5 mg of tissue, shaking by vortex for twenty seconds and, subsequently, centrifuging at 16,000 rpm for five minutes. The supernatants were filtered by 0.22 μM Phenex PTFE filters and analyzed as described below. In all cases, no interfering peaks deriving from the samples were detected. Recovery was assessed by spiking tissue samples with a known amount of ODAF standard solution in methanol at a low, medium and high concentration range, which were subsequently extracted and analyzed as described. Recovery percentages were between 79.741 ± 1.665% and 89.484 ± 1.334%.

##### UHPLC-MS/MS Conditions

The separation was performed on a Luna Omega Polar 50 × 2.1 mm, 1.6 μm (Phenomenex) employing as mobile phases: A) 0.1% HCOOH in H_2_O and B) 0.1% HCOOH in ACN, with the following gradient: zero minutes, 70% B, 0.01–3.00 min, 70–100% B, isocratic for one minute. Then, returning to 70% B in 0.10 min. The flow rate was set at 0.5 mL/min. The column oven was set at 45 °C, and 2 μL of extract was injected. All additives and mobile phases were LCMS grade and purchased from Merck (Milan, Italy). The ESI was operated in positive mode. MS/MS analyses were conducted in scheduled multiple reaction monitoring (MRM), employed as transitions for ODAF: 614.2 > 348.10 (quantifier ion), Q1 pre bias −32.0 V, collision energy: −54.0 V, Q3 pre bias −23.0 V; 614.2 > 349.05 (qualifier ion), Q1 pre bias −32.0 V, collision energy: −53.0 V, Q3 pre bias −16.0 V. Dwell time was 50 msec. The interface temperature, desolvation line temperature, heat block temperature were set to 250 °C, 250 °C and 350 °C, respectively. Nebulizing gas, drying (N_2_) and heating gas (air) were set to 3, 10 and 10 L/min, respectively. ODAF was selected as an external standard for the quantitation. Stock solution (1 mg/mL) was prepared in methanol and the calibration curve was obtained in a concentration range of 0.5–50 ng/mL (R^2^ = 0.9996). Repeatability was established by triplicate injections of sample and solutions at low, medium and high concentration levels of the calibration curve, with the same chromatographic conditions and analyst at the same day and within two consecutive days. This showed good retention and quantitative repeatability with maximum coefficient of variation (CV%) values ≤ 0.06 and 0.40 respectively, whereas inter-day repeatability values were 0.22 and 6.18%. Limits of detection (LOD) and quantification (LOQ) were calculated by the ratio between the standard deviation (SD) and analytical curve slope multiplied by 3 and 10, respectively, obtaining as values: LOD: 0.063 ng/mL; LOQ: 0.210 ng/mL.

##### LN-ODAF Distribution in Ocular Tissues

The ability of LN-ODAF to diffuse through different ocular compartments was tested in vivo, taking advantage of the particles made fluorescent by ODAF. The average diameter of LN-ODAF was estimated to be around 100 nm, allowing sterilization by filtration. For in vivo assays, 3 young adult (3–5 m old) mice belonging to the C57Bl6/J strain, were administered 5 μL of LN-ODAF solution per eye; administrations were repeated three times, waiting five minutes between one and the other. One (control) eye was administered non-fluorescent saline solution, while the others were treated at different times, to achieve exposures of two, four, six and eight hours, respectively. The eyes of two additional mice of the same strain, used for separate studies, were isolated omitting ocular administrations and used as further controls of intrinsic tissue fluorescence. All mice were deeply anesthetized by intraperitoneal injections of Zoletil 100 (80 mg/kg), their eyes were then quickly enucleated and the animals were sacrificed by decapitation. The eyes were left unfixed, quickly immersed in OCT (optimal cutting temperature, Sakura) and embedded in a medium and rapidly frozen in cold isopenthane/dry ice. Consecutive sections (14–18 μm thick) were obtained from each eye with a Leica cryostat at −20 °C and collected on glass slides. Subsequently, the sections were rinsed in phosphate buffer saline (PBS) for ten minutes, left humid, cover slipped and observed with a Zeiss Imager.Z2 microscope equipped with an Apotome 2 device (Zeiss, Milan, Italy) using EC Plan-Neofluar 10×/0.27 and 20×/0.50 M27 objectives. Fluorescence filter settings were 450–490 nm (filter excitation wavelength); 500–550 nm (filter emission wavelength); 495 nm (beam splitter). Adjacent images were obtained systematically and tiled with the appropriate routine of the Zen 3.0 Zeiss program, to reconstruct the profiles of entire eyes. Images brightness and contrast were optimized with Adobe Photoshop CS.

## 5. Conclusions

Although drug delivery to the posterior eye segment is still a great challenge due to the complex eye anatomy, the development of lipid-based nanocarriers (LNs) has made it possible to prolong corneal residence time and improve ocular bioavailability of drugs. In order to investigate how LNs interact with the ocular mucosa and reach the posterior eye segment, we formulated nanocarriers designed to bear a traceable fluorescent probe in the present work. This probe was obtained by a conjugation reaction between fluoresceinamine and the solid lipid excipient stearic acid, forming a stable covalent adduct (ODAF). The fluorescent lipid was then nanostructured to obtain LN-ODAF by solvent-diffusion technique. LN-ODAF were characterized by DLS and TEM analyses, showing technological parameters suitable for ocular administration (particle size around 100 nm). To investigate the ability of LN-ODAF to diffuse through the different ocular compartments, in vivo studies were performed. Microscopy observation showed that the diffusion of LN-ODAF begins at the corneal level (two hours) and spreads to the back of the eye to reach the sclera and the retina (eight hours). From the results obtained, LN-ODAF demonstrated the potential use of lipid-based nanoparticles as efficient carriers of an active pharmaceutical ingredient (API) involved in the treatment of retinal diseases or general posterior eye segment diseases.

## Figures and Tables

**Table 1:** Mean particle size (Z-Ave), polydispersity index (PDI) and zeta potential (ZP) of LN-ODAF.

Formulation	Z-Ave [nm ± SD]	PDI [–] ± SD	ZP [mV ± SD]
LN-ODAF	112.7 ± 0.4	0.249 ± 0.3	−22.1 ± 0.6

**Table 2:** Stability of ODAF at different pHs. Results are expressed as percentage of peak area in comparison with the standard solutions at the same concentrations.

Time Point		Area %	
	pH 5	pH 7	pH 9
2h	97.61% ± 0.60	97.79% ± 0.55	112.40% ± 0.24
4h	99.21% ± 0.05	99.21% ± 0.10	127.27% ± 0.77

**Table 3:** Quantitative determination of ODAF in samples. Results are expressed as ng of ODAF for mg of extracted tissue ± standard deviation (SD). (LOD: 0.063 ng/mL; LOQ: 0.210 ng/mL).

Tissue	Untreated Ctrl	1 h	3 h	8 h	16 h
Aqueous humor	ND	0.5890 ± 0.0268	ND	ND	ND
Vitreous humor	ND	0.3290 ± 0.0174	0.1215 ± 0.017	ND	ND
Cornea	ND	0.1090 ± 0.0078	0.0857 ± 0.013	0.1010 ± 0.021	0.1089 ± 0.011
Iris	ND	0.2365 ± 0.0113	0.0915 ± 0.004	0.0947 ± 0.009	ND
Crystalline lens	ND	<LOD	0.1805 ± 0.037	0.0931 ± 0.019	0.0926 ± 0.037
Retina	ND	0.3730 ± 0.0277	0.2190 ± 0.067	0.0941 ± 0.012	0.0891 ± 0.006
Sclera	ND	0.2672 ± 0.0095	0.7055 ± 0.088	0.4717 ± 0.054	0.158 ± 0.027

## Data Availability

The data presented in this study are available on request from the corresponding author.

## Supplementary Materials

The following are available online. Figure S1: ^1^H-NMR (500 MHz, DMSO-d_6_) spectrum of ODAF. Figure S2: ^13^C-NMR (126 MHz, DMSO-d_6_) spectrum of ODAF. Figure S3: gHSQCAD spectrum of ODAF.

## Sample Availability

Samples of the compounds are not available from the authors.
