# Supplementary material for: Lipid Nanoparticles Traverse Non-Corneal Path to Reach the Posterior Eye Segment: In Vivo Evidence
Source: Molecules. 2021 Aug 2;26(15):4673. doi: 10.3390/molecules26154673 (PMC8347557; doi:10.3390/molecules26154673)

## Supporting Information

**Figure S1**. $^{1}$H-NMR (500 MHz, DMSO-d6) spectrum of ODAF.

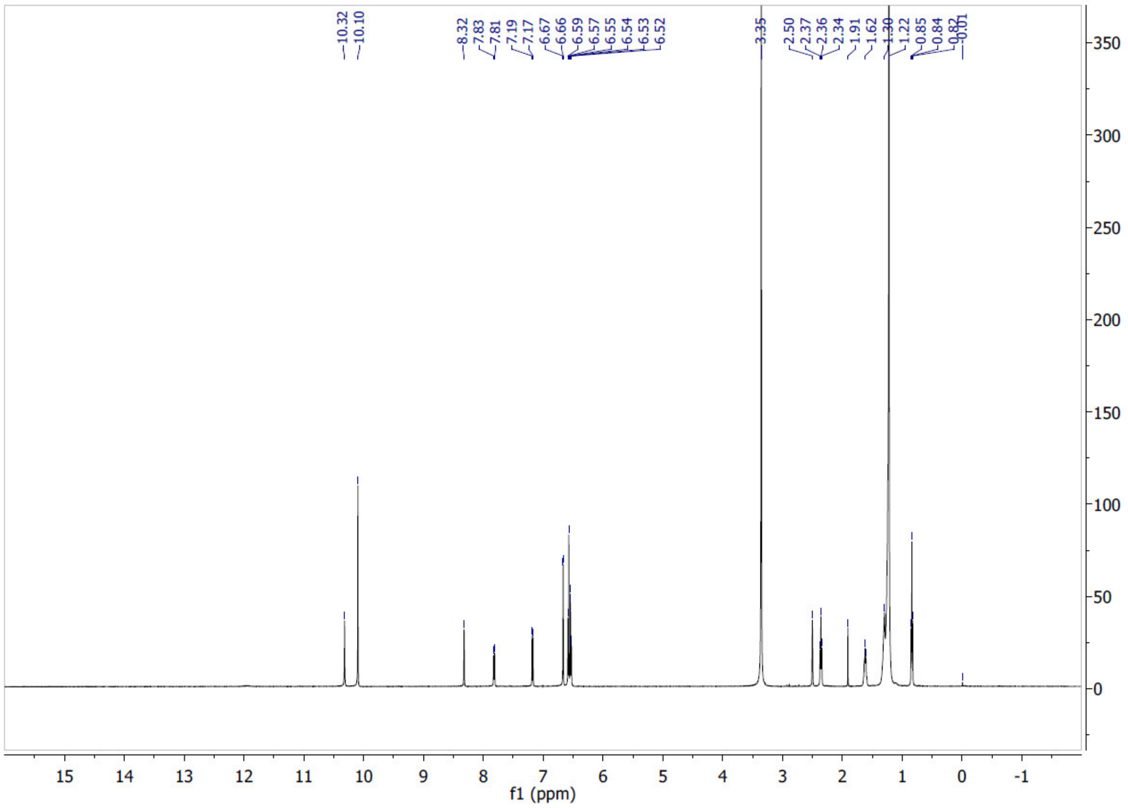


**Figure S2**. $^{13}$C-NMR (126 MHz, DMSO-d6) spectrum of ODAF.

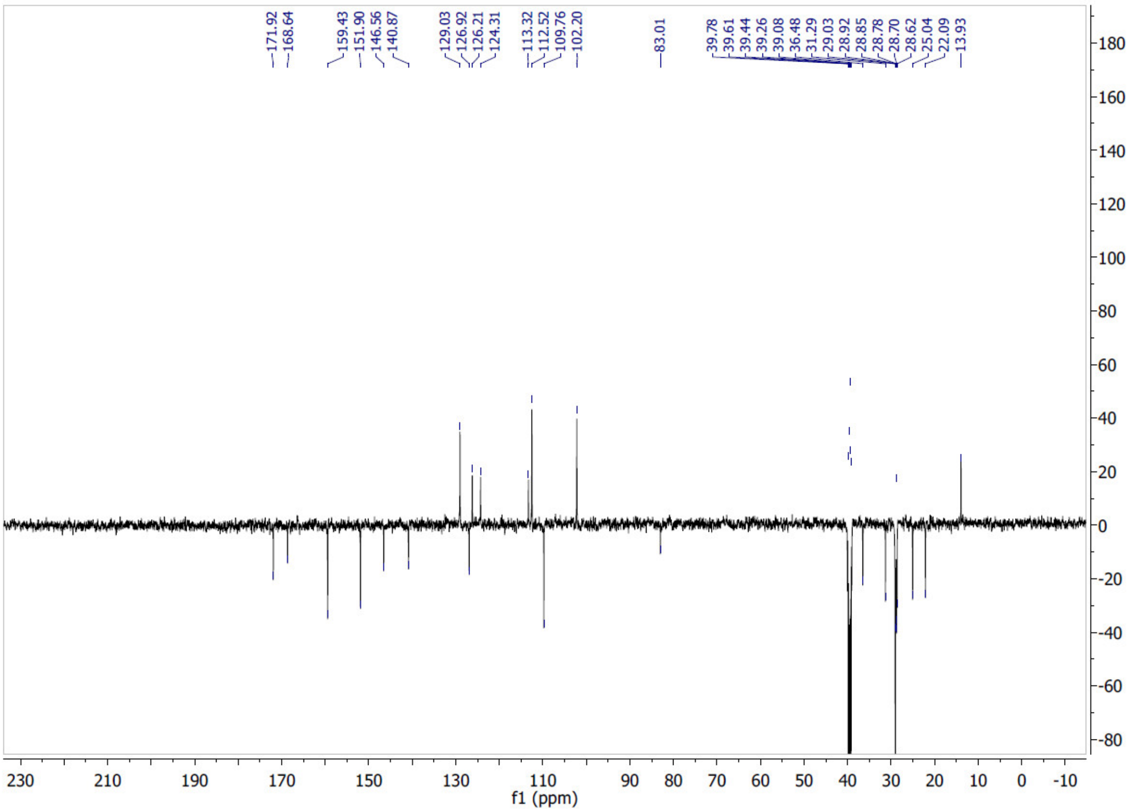

**Figure S3**. gHSQCAD spectrum of ODAF.

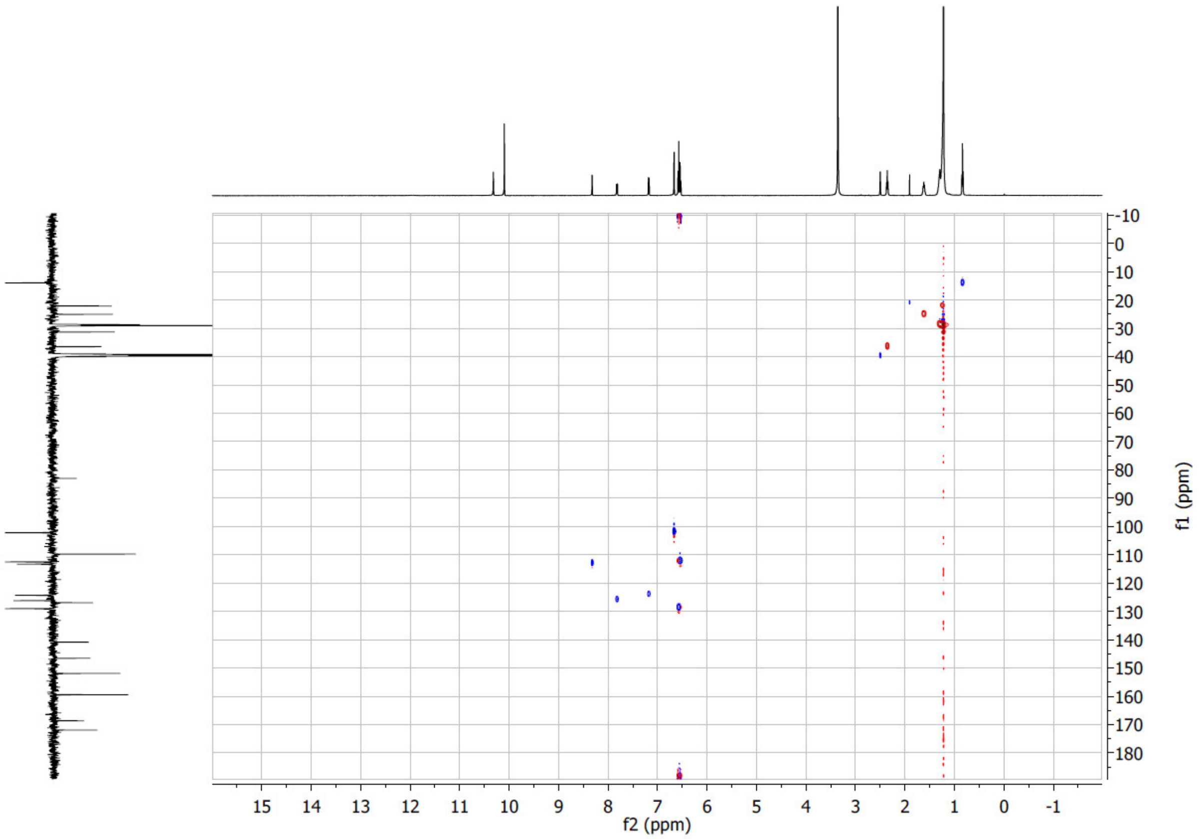

Supplement: Supplementary file 1 [file molecules-26-04673-s001.zip › molecules-1316393-supplementary.pdf]
